# Novel patient-derived xenograft mouse model for pancreatic acinar cell carcinoma demonstrates single agent activity of oxaliplatin

**DOI:** 10.1186/s12967-016-0875-z

**Published:** 2016-05-10

**Authors:** Jason C. Hall, Laura A. Marlow, Adam C. Mathias, Louis K. Dawson, William F. Durham, Kenneth A. Meshaw, Robert J. Mullin, Aidan J. Synnott, Daniel L. Small, Murli Krishna, Daniel von Hoff, Julia Schüler, Steven N. Hart, Fergus J. Couch, Gerardo Colon-Otero, John A. Copland

**Affiliations:** Department of Cancer Biology, Mayo Clinic Comprehensive Cancer Center, 4500 San Pablo Road S., Jacksonville, FL 32224 USA; Department of Laboratory Medicine and Pathology, Mayo Clinic, 4500 San Pablo Rd S., Jacksonville, FL 32224 USA; Division of Hematology/Oncology, Mayo Clinic, 4500 San Pablo Rd S., Jacksonville, FL 32224 USA; Charles River Discovery Services, 3300 Gateway Centre Blvd., Morrisville, NC 27560 USA; The Translational Genomics Research Institute (TGen), 445 N 5th St., Phoenix, AZ 85004 USA; Department of Laboratory Medicine and Pathology, 200 First Street SW, Rochester, MN 55905 USA

**Keywords:** Oxaliplatin, Patient derived tumor xenograft, Tumor, Pancreatic acinar cell carcinoma, Chemotherapy, Individualized medicine, Precision medicine, BRCA2

## Abstract

**Background:**

Pancreatic acinar cell carcinoma (PACC) is a rare malignancy, accounting for <1 % of all pancreatic neoplasms. Very few retrospective studies are available to help guide management. We previously reported the case of a patient with metastatic PACC who achieved prolonged survival following doxorubicin treatment. Personalized treatment was based on molecular and in vitro data collected from primary cells developed from their liver metastasis. We now report the characterization of a patient derived tumor xenograft (PDTX) mouse model that originated from this patient’s PACC liver metastasis.

**Methods:**

Fragments of biopsy tissue (5 mm^3^) from PACC liver metastasis were implanted into athymic nude mice. Tumors were grown and passaged from the host mice into new mice to be tested for therapeutic response. Immuno-histochemical (IHC) biomarkers were used to confirm that the PDTX model represents human PACC. The antitumor activities of multiple drugs (5-FU, irinotecan, oxaliplatin, gemcitabine, bevacizumab, erlotinib, doxorubicin and imatinib) were tested. Tumor size was measured over 74 days or until they reached an endpoint volume of ~800 mm^3^. Tests to measure serum lipase levels and histological analyses of tumor tissues were also conducted to assess PACC progression and re-differentiation.

**Results:**

The model presented here expresses the same IHC markers found in human PACC. In the chemotherapy study, oxaliplatin produced a prolonged durable growth response associated with increased apoptosis, decreased serum lipase levels and increased healthy acinar cells. Bevacizumab also produced a significant growth response, but the effect was not prolonged as demonstrated by oxaliplatin treatment. The other chemotherapies had moderate to little effect, particularly after treatment ceased. Mutations in DNA repair genes are common in PACC and increase tumor susceptibility to oxaliplatin. To explore this we performed IHC and found no nuclear expression of BRCA2 in our model, indicating a mutation affecting nuclear localization. Gene sequencing confirms BRCA2 has a homozygous gene deletion on Exon 10, which frequently causes a protein truncation.

**Conclusions:**

In summary, we report the development and characterization of the first and only preclinical PACC PDTX model. Here we show sustained anti-tumor activity of single agent oxaliplatin, a compound that is more effective in tumors that harbor mutations in DNA repair genes. Our data shows that BRCA2 is mutated in our PACC model, which could contribute to the oxaliplatin sensitivity observed. Further studies on this rare PACC model can serve to elucidate other novel therapies, biomarkers, and molecular mechanisms of signaling and drug resistance.

**Electronic supplementary material:**

The online version of this article (doi:10.1186/s12967-016-0875-z) contains supplementary material, which is available to authorized users.

## Background

Pancreatic acinar cell carcinoma (PACC) is a rare, frequently lethal disease, accounting for less than 1 % of all pancreatic neoplasms tumors [1–4]. A retrospective series of 672 patients with PACC reported a 47 months median survival [5]. A review of patients seen at Memorial Sloan Kettering from 1981 to 2001 found that the median survival of patients with metastasis was 14 months as compared to 38 months in the absence of metastasis [1]. A follow up retrospective study reviewing patients seen from 2000 to 2011 found a median survival of 57 months for patients with localized resected tumors and a 19 months median survival in patients with metastatic disease [6].

Patients with PACC frequently present with abdominal pain and bloating as the dominant symptoms, and in some cases their initial clinical diagnosis given was acute pancreatitis [1, 7, 8]. Elevation of serum lipase levels can be associated with systemic fat necrosis, a significant cause of morbidities in PACC patients [9–12]. However, elevated serum lipase and amylase levels can be seen in both PACC and acute pancreatitis leading to difficulties in establishing a correct diagnosis [13, 14]. Reliable markers of PACC have been slowly emerging such as carcinoembryonic antigen (CEA) [15], cytokeratin 18 (CK18) [16] and B-cell lymphoma/leukemia 10 (BCL10) [17, 18] allowing us to distinguish acinar cell carcinoma from normal acinar cells or other pancreatic cancers through histology.

The genetic and molecular abnormalities that lead to PACC have not been fully identified. Recent studies have found that genes regulating DNA repair may be mutated in PACC patients. DNA repair mutations were noted in 45 % of PACC tumors, BRCA2 being the most common gene, followed by BRCA1 and ATM20 [3, 6, 19]. Previously, we reported on a case of a PACC patient receiving personally designed treatments based on the genetic and molecular profiles of his tumor [20]. We cultured the patient’s tumor biopsies into primary cell lines and treated them with different chemotherapy agents [20]. The outcome was that a DNA replication inhibitor, (irinotecan), a DNA intercalating agent and transcription inhibitor (doxorubicin), and a tyrosine kinase inhibitor (imatinib) were among the most effective agents against his tumor cells in cell culture [20]. As a result of the observations seen in vitro, liposomal doxorubicin was administered and the patient had evidence of a sustained clinical response throughout the treatment regimen [20].

Here, we report the characterization of a PACC patient derived tumor xenograft (PDTX) mouse model (PA-018) from the patient’s tumor biopsy. With the use of PA-018, multiple chemotherapies (5-FU, oxaliplatin, gemcitabine, liposomal doxorubicin) and targeted agents (irinotecan, bevacizumab, erlotinib, imatinib) were tested in vivo. Of the therapies tested, oxaliplatin was the most promising and demonstrated sustained antitumor activity after only 3 weekly treatments. Therefore, evaluation of potential effective treatments using this PDTX model is a viable technology that may facilitate the discovery of effective treatments against this rare tumor. We provide the first human derived PACC tumor model now available to the PACC scientific community.

## Methods

### Development of PDTX model

Biopsy tissue from PACC liver metastasis was implanted subcutaneously into two 5 week old anesthetized athymic nude female mice strain #069 (Harlan Laboratories, Indianapolis, IN) under IACUC approved procedures. Implanted tumors were harvested and frozen as 5 mm^3^ fragments in 10 % DMSO-DMEM media and multiple “passages” were continued in athymic nude mice. Applying this technique to the patient’s tumor has created a renewable source of PACC tissue and a representative in vivo model to test promising drugs. This PACC PDTX model is available at Charles Rivers (CR) Discovery Services (Morrisville, NC).

### In vivo implantation and tumor growth

For the in vivo study, 6 week old female athymic nude mice strain #490 (CR Discovery Services) were subcutaneously implanted with 5 mm^3^ tumor fragments into the right flanks. They were fed ad libitum NIH 31 Modified and Irradiated Lab Diet^®^ consisting of 18.0 % crude protein, 5 % crude fat, and 5 % crude fiber and housed on irradiated Enrich-o’cobs™ Laboratory Animal Bedding in static microisolators on a 12-hour light cycle at 20–22 °C (68–72 °F) and at 40–60 % humidity. CR Discovery Services specifically complies with the recommendations of the *Guide for Care and Use of Laboratory Animals* with respect to restraint, husbandry, surgical procedures, feed and fluid regulation, and veterinary care. The animal care and use program at CR Discovery Services is accredited by the Association for Assessment and Accreditation of Laboratory Animal Care International, which assures compliance with accepted standards for the care and use of laboratory animals.

Fifty-five days later (~14 weeks old), designated as day 1 of the study, mice were sorted into treatment groups with individual tumor volumes ranging from 75 to 245 mm^3^ and group mean tumor volumes of 164–170 mm^3^. Tumor size, in mm^3^, was calculated from:

$${\text{Tumor}}\,{\text{Volume}} = \frac{{w^{2} \, \times \,l}}{2}$$

where *w* is the width and *l* is the length, in mm, of the tumor. Tumor weight was estimated with the assumption that 1 mg is equivalent to 1 mm^3^ of tumor volume.

### Test articles and dosing regimens

The following therapies were prepared on each day of dosing as follows: 5-Fluorouracil or 5-FU (TEVA Pharmaceuticals, 50 mg/mL, Lot# 6102655) was diluted to 10 mg/mL with sterile 5 % dextrose in water (D5 W) and administered at 100 mg/kg intra-peritoneal (ip) weekly for 3 weeks. Irinotecan solution for injection (Sandoz Pharmaceuticals, Inc., 20 mg/mL, Lot# CF0165) was diluted to 10 mg/mL with D5 W and administered at 100 mg/kg ip once weekly for 3 weeks. An aliquot of oxaliplatin stock (Eloxatin^®^, Sanofi Aventis, 5 mg/mL, Lot# CH630) was diluted to 1 mg/mL with sterile D5 W, which provided a 10 mg/kg dosage that was administered ip once weekly for 3 weeks. Gemcitabine (Gemzar^®^, Eli Lilly, Lot# A906313D) was reconstituted to 12 mg/mL with sterile saline (0.9 % NaCl) and 120 mg/kg was administered ip daily every 3 days for a total of four doses. An aliquot of Bevacizumab stock (Avastin^®^, Genentech, Lot# 956583, 25 mg/mL) was diluted to 0.5 mg/mL with saline, which provided a 5 mg/kg dosage that was administered ip twice weekly for five weeks. Liposomal doxorubicin (Doxil, Sequus Pharmaceuticals, Inc., 2 mg/mL, Lot# 1107161) was diluted to 0.3 mg/mL in saline, which provided a 3 mg/kg dosage that was administered intravenously (i.v.) once weekly for 3 weeks. Imatinib mesylate (Gleevec^®^, Novartis Pharmaceuticals Corp, 100 mg tablets) dosing suspensions were prepared by resuspending the required amount of tablets in sterile water for injection to yield a final concentration of 10 mg/mL and then administered orally (po) at 100 mg/kg once daily for 28 days. Dosing suspensions of erlotinib (Tarceva^®^, OSI Pharmaceuticals, Inc., Lot# 1121701CW, 100 mg tablets) were prepared by resuspending the required amount of tablets in 1 % CMC : 0.1 % Tween80 in sterile water to produce a final concentration of 8 mg/mL and then administered orally at of 80 mg/kg once daily for 15 days. In all groups, the dosing volume of 10 mL/kg (0.2 mL/20 g mouse) was scaled to the weight of each individual animal.

### Endpoint

Tumors were measured twice weekly using calipers. Each animal was euthanized when its neoplasm reached the endpoint volume of 800 mm^3^ or day 74, whichever came first. The time-to-endpoint (TTE) for each mouse was calculated for each endpoint by the following equation:

$${\text{TTE}} = \frac{{\log_{10} ({\text{endpoint}}\,{\text{volume}}) - {\text{b}}}}{\text{m}}$$

where TTE is expressed in days, endpoint volume is expressed in mm^3^, b is the intercept, and m is the slope of the line obtained by linear regression of a log-transformed tumor growth data set. The data set is comprised of the first observation that exceeded the endpoint volume used in analysis and the three consecutive observations that immediately preceded the attainment of this endpoint volume. Any animal that did not reach endpoint was euthanized at the end of the study and assigned a TTE value equal to the last day of the study. Any animal determined to have died from treatment-related (TR) causes was to be assigned a TTE value equal to the day of death. Any animal that died from non-treatment-related (NTR) causes was excluded from the analysis.

Treatment efficacy was determined from tumor growth delay (TGD), which is defined as the increase in the median TTE for a treatment group compared to the control group:

$${\text{TGD}} = {\text{T}} - {\text{C,}}$$

expressed in days, or as a percentage of the median TTE of the control group:

$$\% {\text{TGD}} = \frac{{{\text{T}} - {\text{C}}}}{\text{C}} \times 100$$

where T is the median TTE for a treatment group, C is the median TTE for control Group 1.

### Toxicity

Test animals were observed frequently for any overt signs of adverse, treatment-related side effects, and clinical signs of toxicity were recorded. Test animals were also weighed twice weekly and any animal that exceeded the limits for acceptable body weight (BW) loss was euthanized. Dosing was suspended in any group that exceeded the limits for acceptable mean BW loss. If mean BW recovered, then dosing may be resumed in that group, but at a lower dosage or less frequent dosing schedule. Acceptable toxicity for the maximum tolerated dose (MTD) was defined as group mean BW loss of less than 20 % during the test, and no more than 10 % TR mortality. A death was classified as TR if it was attributable to treatment side effects as evidenced by clinical signs and/or necropsy, or due to unknown causes during the dosing period or within 14 days of the last dose. A death was classified as NTR if there is no evidence that the death was related to treatment side effects.

### Statistical analysis and graphical presentations

Prism (GraphPad) for Windows 6.02 was used for all statistical analysis and graphical presentations. The logrank test was employed to assess the significance of the difference between the overall survival experiences of two groups. The logrank test analyzed the individual TTEs for all animals in a group, except those lost to the study due to NTR deaths. The two-tailed statistical analysis was conducted at *P* = 0.05. Prism reports results as non-significant (ns) at *P* > 0.05, significant (symbolized by “*”) at 0.01 < *P* ≤ 0.05, very significant (“**”) at 0.001 < *P* ≤ 0.01 and extremely significant (“***”) at *P* ≤ 0.001. Since the logrank test is a test of significance and does not provide an estimate of the size of the difference between groups, all levels of significance are reported as either significant or non-significant within the context of this report. When an animal exited the study due to tumor size or TR death, the final tumor volume recorded for the animal was included with the data used to calculate the median volume at subsequent time points. Tumor growth curves were truncated when the tumors in more than 50 % of the assessable animals in a group have grown to the endpoint volume or treatment exceeded the MTD (≥20 % body weight loss or >10 % TR related mortality).

### Serum lipase

Blood was collected sublingually under no anesthesia at pre-dose and day 15 for n = 5 mice per group in EDTA collection tubes. Serum was collected and analyzed by IDEXX laboratories (Westbrook, Maine) for serum lipase levels.

### Immunohistochemistry (IHC) and immunofluorescence (IF)

Formalin-fixed tissues were collected and embedded into paraffin. A tissue microarray (TMA) was constructed for all tumor tissues from each of the treatment groups and used for IHC analysis. TMA tissues were cut into 5 mm sections, deparaffinized, hydrated, antigen retrieved and blocked with diluent that contained Background Reducing Components (Dakocytomation, Denmark). Immunostaining was done on either the TMA or PA-018 alone with the following: carcinoembryonic antigen (CEA), neuron specific enolase (NSE), Chromogranin A (CgA), and cytokeratin 19 (CK19) [1:100, anti-mouse with rodent block (Dakocytomation)]; Mist-1 [1:2000] and cleaved caspase-3 (CC3) [1:100 hi pH, anti-rabbit (Cell Signaling, Beverly, MA)]; amylase [1:1000, anti-rabbit (Sigma-Aldrich, St.Louis, MO)]; lipase [1:1600], BRCA1 [1:100], and Collagen I [1:1500, anti-rabbit (Abcam, Cambridge, MA)]; BCL-10 [1:200, anti-rabbit] and CD31 [1:100, anti-goat (Santa Cruz, CA)]; BRCA2 [1:500, anti-mouse (R&D systems)]. For IHC the Envision Dual Labeled Polymer kit (DakoCytomation) was used according to the manufacturer’s instructions and then lightly counterstained with Gill I hematoxylin (Sigma-Aldrich) before dehydration and mounting. Images were obtained using Scanscope XT (Aperio Technologies, Vista, CA) and the staining of the TMA punches were scored using an algorithm in the Imagescope software (Aperio Technologies) created by a histologist based upon signal intensity (0, 1+ , 2+ , 3+) and percentage. For immunofluorescence, tissue was incubated with AlexaFlour 594-conjugated secondary antibody (Invitrogen) and mounted with Vectashield containing DAPI (Vector Laboratories, Burlingame, CA). Images were acquired on a Zeiss LSM 880 confocal laser scanning microscope (Carl Zeiss MicroImaging, Inc, Thornwood, NY).

### Gene mutation analysis

DNA from a fresh frozen sample of PA-018 was isolated and underwent custom capture (eArray; Agilent, Santa Clara, CA) of the coding region and intron/exon boundaries of coding exons for BRCA2. Products from each capture reaction were sequenced on a HiSeq 2000 (Illumina, San Diego, CA) and analyzed as described by Couch et al. [21].

## Results

### Characterization of the PACC PDTX mouse model

**Fig. 1:**
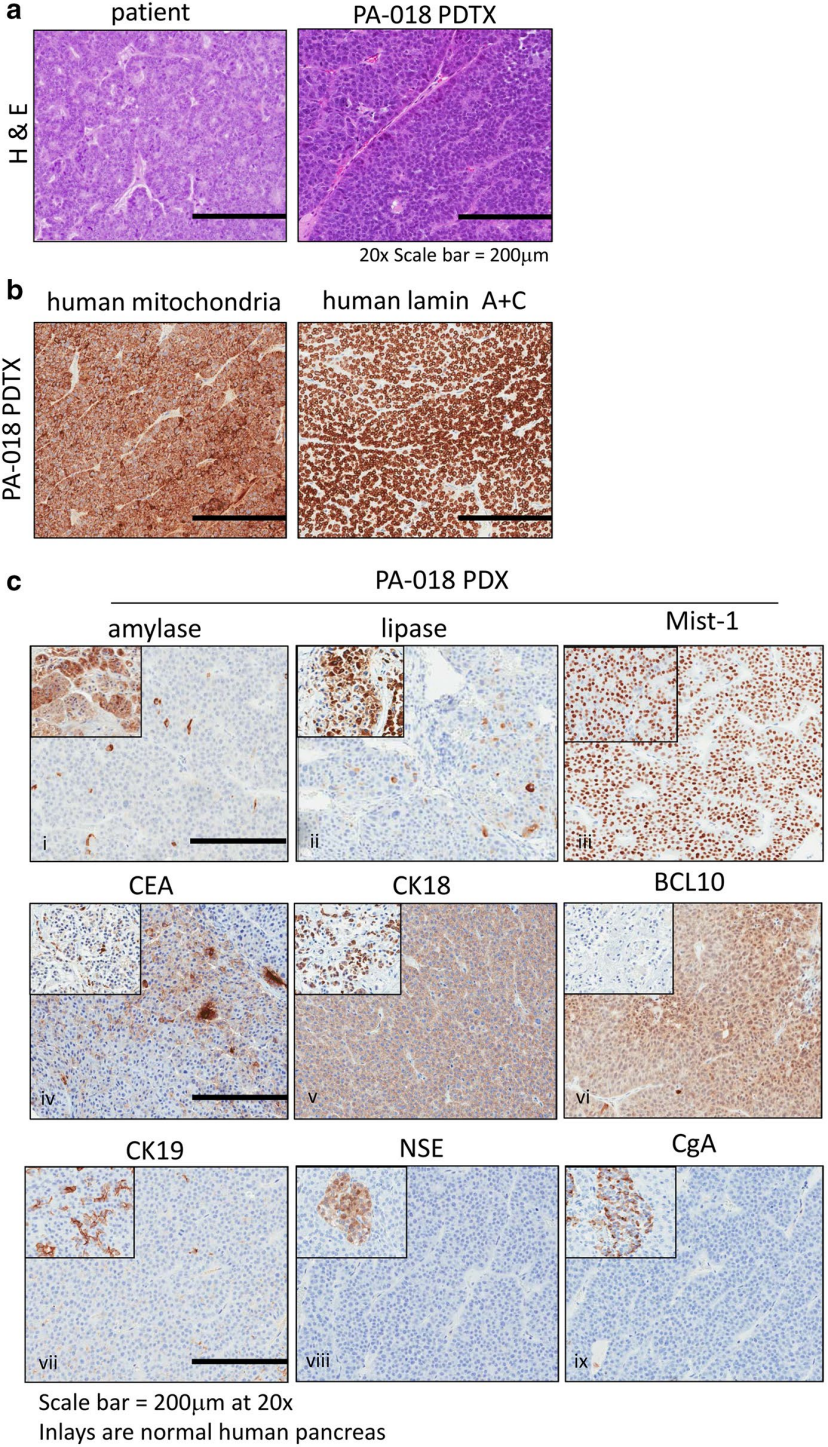
Characterization of the PACC PDTX mouse model. **a** Hemotoxylin and eosin (H&E) staining of paraffin embedded patient and PDTX PACC tumor tissues (magnification X20) **b** Immunohistochemistry (IHC) of human specific antibodies, mitochondrial surface marker and lamin A+C. **c** IHC for acinar cell markers [amylase, lipase, Mist-1], acinar cell carcinoma markers [carcinoembryonic antigen (CEA), cytokeratin 18 (CK18), B-cell lymphoma/leukemia 10 (BCL10)], ducts [cytokeratin 19 (CK19)] and neuroendocrine markers [neuron specific enolase (NSE), chromogranin A (CgA)] at X20 magnification. Inlays are normal human pancreas

PA-018, a pancreatic acinar cell carcinoma (PACC) PDTX mouse model, was derived from a metastatic liver biopsy of a patient previously described in a case report [20]. The histologic features of PA-018 were similar to those in the patient’s pancreatic primary tissue (Fig. 1a). These features included a solid, acinar-like growth pattern by cells with undifferentiated nuclei and amphophilic cytoplasm. Immunohistochemistry (IHC) with human specific mitochondrial surface protein and human lamin A+C and confirmed that the xenograft tumor cells were of human origin (Fig. 1b). Short tandem repeat (STR) analysis showed that the genetic signature of our PDTX tumor (passage 5) closely matched (allele drop out was noted) the signature of the patient. The DNA that was used for comparison was from a sample taken 3 years prior to the PA-018 biopsy due to unavailable tissue (Additional file 1: Table S1).

IHC characterization of PA-018 showed a reduction in cytoplasmic expression of amylase and lipase as compared to the normal human pancreas, while the pancreatic acinar marker, Mist-1 [22], was widely expressed in the tumor tissue (Fig. 1c, panels i–iii). PA-018 also expressed previously documented markers of PACC, including CEA [15], CK18 [16] and BCL10 [17, 18] (Fig. 1c, panels iv, vi) and had a lack of cytokeratin 19 (CK19) expression, a marker of ducts and pancreatic ductal tumors [23] (Fig. 1c, panel vii). Since PACC can have heterogeneous tumor populations, which include neuroendocrine derived cancer cells, we stained for neuroendocrine markers, such as neuron specific enolase (NSE) and chromogranin A (CgA). PA-018 was negative for NSE and CgA as compared to the islet cells in normal human pancreas [24] (Fig. 1c, panels viii–ix).

### Evaluation of different chemotherapies demonstrate that oxaliplatin sustains tumor growth inhibition in the PACC PDTX mouse model

**Fig. 2:**
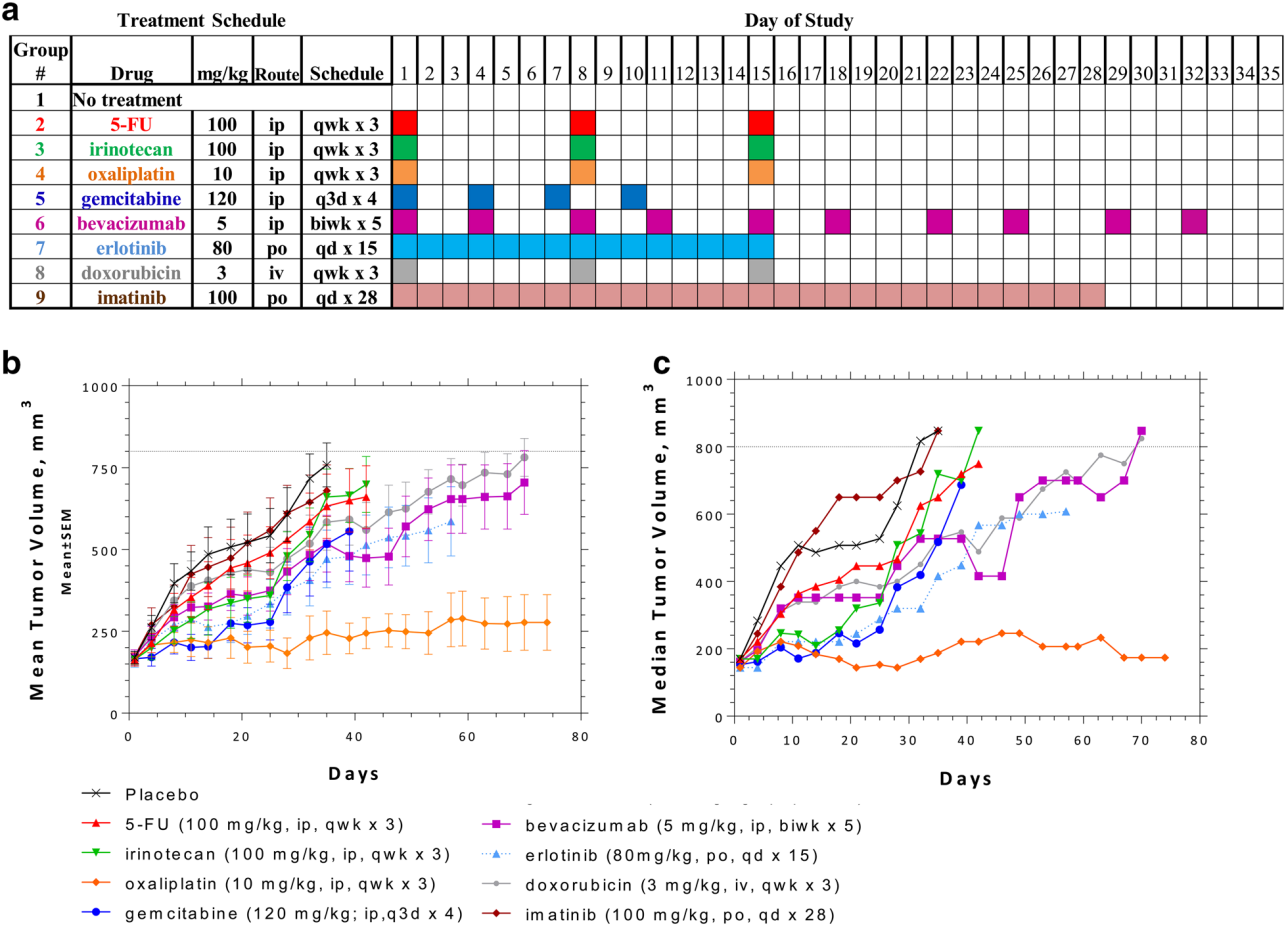
Therapeutic response in PACC PDTX mouse model. **a** Reference table for chemotherapy doses along with route and treatment schedule. **b** Groups 1 to 9 mean volumes ± standard error (SEM) were plotted as a function of time. **c** Groups 1 to 9 median tumor volumes were plotted as a function of time. Tumor growth was continually observed after treatment regimen ceased. When an animal exited the study due to tumor size, the final tumor volume recorded for the animal was included with the data to calculate the mean and median volume at subsequent time points as indicated by *dashed lines*

For studies on the effects of different monotherapies, PA-018 was expanded and 4 mm^3^ tissues (passage 5) were ectopically implanted into flanks of athymic nude female mice. Therapy was implemented once tumors reached an average of 100 mm^3^. Agents tested included; DNA synthesis inhibitors (5-FU, gemcitabine), a DNA alkylating agent (oxaliplatin), a DNA intercalating agent (liposomal doxorubicin), a topoisomerase inhibitor (irinotecan), an EGFR inhibitor (erlotinib), a c-kit inhibitor (imatinib) and an angiogenesis inhibitor (bevacizumab). The rationale for these drug choices were based upon previous gene array and protein analyses performed on the patient tumor tissue [20]. Possible drug therapies and interactions with upregulated genes include: CES2 (irinotecan), TOP2B (liposomal doxorubicin), and DNA repair genes (oxaliplatin). Proteins upregulated include EGFR (erlotinib), and c-kit (imatinib) [20]. Treatment schedules along with doses, route and frequencies are summarized in Fig. 2a.

Schedules and dosing represent a blend of the maximally effective dose and/or maximally tolerated dose based on several experiments conducted by Charles River Discovery. Doses are in a range that reflects preclinical studies that have been seen previously in the literature [25–33].

Tumor growth was monitored individually even after therapy regimen was concluded and allowed to grow to an endpoint of 650–800 mm^3^or up to 74 days. This strategy tested the therapy durability of the antitumor activity in order to determine “time-to-endpoint” (TTE). A group was terminated once the tumor burden reached endpoint or if fewer than 50 % of the assessable animals in a group remained on study. When individual mice were exited from the study due to “treatment-related” (TR) deaths, the final tumor volume recorded for the animal was included with the data to calculate the mean and median tumor volume at subsequent time points as indicated by dashed lines seen in Fig. 2b, c. Tumor growth volumes were excluded for deaths assessed as “non-treatment related” (NTR). Sample size (n) was adjusted in the event of NTR deaths (Fig. 2; Table 1).

**Table 1:** Result summary of PA-018 therapeutic responses and toxicities

Group	N	Treatment regimen				Median		Statistical significance			Mean BW	Deaths	
		Agent	mg/kg	Route	Schedule	TTE	TGD (T-C)	Chi square	P value	Summary	Nadir	TR	NTR
1	10	placebo	–	–	–	32.7	–	–	–	–	−0.1 % day 14	0	0
2	10	5-FU	100	ip	qwk × 3	37.3	4.6	0.3966	0.5288	ns	−5.9 % day 21	1	0
3	10	irinotecan	100	ip	qwk × 3	40.8	8.1	0.5419	0.4616	ns	−7.9 % day 21	0	0
4	10	oxaliplatin	10	ip	qwk × 3	74*	41.3*	14.82	*0.0001*	***	−9.8 % day 21	0	0
5	10	gemcitabine	120	ip	q3d × 4	37.8	5.1	1.265	0.2607	ns	−5.2 % Day 39	1	0
6	9	bevacizumab	5	ip	biwk × 5	*68.8*	*36.1*	4.165	*0.0413*	^a^	−4.3 % day 21	0	1
7	9	erlotinib	80	po	qd × 15	54.4	*21.7*	1.277	0.2585	ne	−*15.3* *% day 14*	2	1
8	10	doxorubicin	3	iv	qwk × 3	65.8	*33.1*	2.525	0.112	ns	−5.1 % day 42	0	0
9	9	imatinib	100	po	qd × 28	33.3	0.6	0.09389	0.7593	ns	−0.8 % day 21	0	1

The therapies used included DNA synthesis inhibitors (5-FU, gemcitabine), a DNA alkylating agent (oxaliplatin), a DNA intercalating agent (liposomal doxorubicin), a topoisomerase inhibitor (irinotecan), an EGFR inhibitor (erlotinib), a c-kit inhibitor (imatinib) and an angiogenesis inhibitor (bevacizumab). Therapies were delivered as indicated and tumor growth was continually observed after treatment regimen ceased in order to determine time-to-endpoint (TTE) and difference between median TTE of treated groups vs. placebo (T-C). Statistical significance was evaluated by logrank test, df = 1 with significance indicated by * and non-significance (ns) or not evaluable (ne). Body weight (BW) nadir was shown as percent change and deaths were divided into treatment-related deaths (TR) and non-treatment related deaths (NTR). The final sample size (n) was calculated by removing NTR deaths

*n* number of animals in a group not dead from accidental or unknown causes, or euthanized for sampling, *TTE* time to endpoint, *T*-*C* difference between median TTE (days) of treated group versus control group, *TR* treatment-related death, *NTR* non-treatment-related death, *Mean BW Nadir* lowest group mean body weight, as  % change from day 1, *ne* not evaluable, *ns* not significant

Statistical significance (Logrank test, df = 1): * P < 0.05, ** P < 0.01, *** P < 0.001, compared to Group 1

^a^Time of sacrifice (74 days) was artificially used at TTE for oxaliplatin group

Plotting both mean and median tumor volume for each treatment group revealed that imatinib had no effect on tumor growth with a TTE of 33.3 days as compared to placebo with a TTE of 32.7 days. T-C was only 0.6, which is the “difference between the median TTE of treated group vs. placebo control”. Irinotecan (T-C = 8.1), gemcitabine (T-C = 5.1), and 5-FU (T-C = 4.6) maintained a strong response while on therapy, but the tumor rapidly grew once the drug was discontinued. Erlotinib (T-C = 21.7), bevacizumab (T-C = 36.1) and liposomal doxorubicin (T-C = 33.1) maintained an intermediate response during therapy and tumor volume slowly increased over time. By far, the most effective agent was oxaliplatin which sustained a strong anti-tumor response for the entire course of this study (74 days) with only 3 weekly treatments (Fig. 2; Table 1). Statistical significance was evaluated by log rank test and overall both oxaliplatin (P = 0.0001) and bevacizumab (P = 0.0413) treatments demonstrated significant anti-tumor activity while liposomal doxorubicin was considered trending (P = 0.112). Erlotinib was also considered trending, but the data was not evaluable due to nadir from toxicities with body weight (BW) percent change of −15.3 % and 2 TR deaths (Table 1).

### Serum lipase levels correlate with response to chemotherapy

**Fig. 3:**
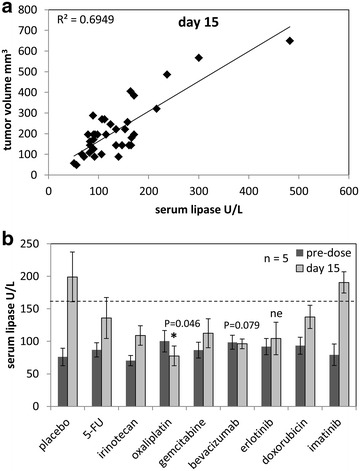
Evaluation of serum lipase enzyme levels. **a** Correlation plot of individual serum lipase levels compared to tumor volume indicated positive correlation with R^2^ = 0.6949 at day 15 when all groups were actively receiving chemotherapeutics. **b** Serum analysis for lipase secretion was performed on blood collected at pre-dose, and day 15. Data was plotted as mean ± standard deviation, n = 5. *Asterisk* indicated P < 0.05 for treatment group compared to placebo, n = 5. Erlotinib was not evaluable (ne) due to n = 3

Serum lipase levels were monitored on the blood of the xenografts to examine pancreatic lipase secretion compared to tumor volume [20]. On day 15, a positive correlation among the groups between serum lipase levels and tumor volumes in the xenografts was observed with R^2^ = 0.6949 (Fig. 3a). Among the groups, lipase secreted into serum (day 15) was only able to significantly decrease following oxaliplatin treatment (P = 0.046), reaching levels below pre-treatment. Bevacizumab treatment also led to lower serum lipase levels as compared to placebo, but it was not significant (P = 0.079). Of the eight monotherapies, only 5-FU and imatinib were unable to decrease serum lipase levels as compared to the placebo control (Fig. 3b).

### Endpoint therapeutic evaluation of proliferation, tumor vascularity, and apoptosis shows that oxaliplatin induces cell death in PACC

**Fig. 4:**
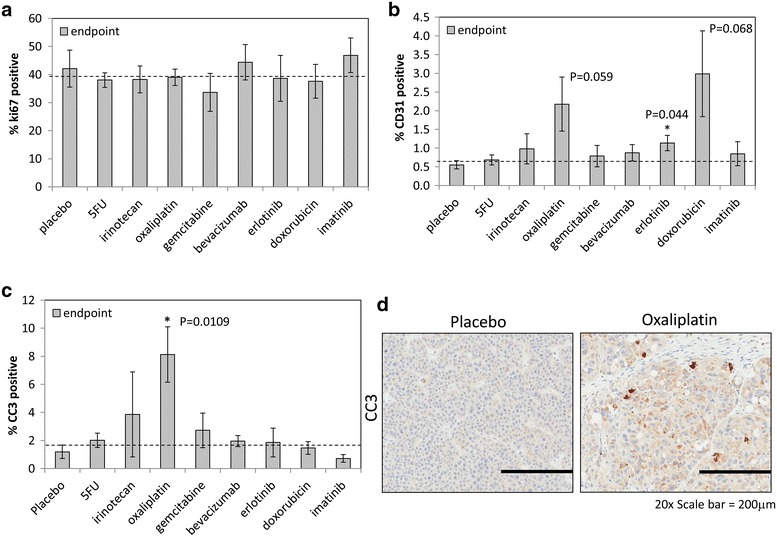
Endpoint therapeutic effects on proliferation, tumor vascularity, and apoptosis. **a** Ki67 for proliferation index was scored by positive counts per core section and plotted as mean percent positive ± standard deviation with no change observed at endpoint. **b** CD31 for blood vessel density was scored by positive pixel count over area and plotted as mean percent ± standard deviation, only erlotonib had a significant change observed at endpoint (P = 0.44). **c** Cleaved caspase-3 (CC3) for apoptotic index was scored by positive pixel count over area and plotted as mean percent ± standard deviation. Oxaliplatin treatment yielded significant apoptosis (P = 0.0109) compared to placebo. **d** Representative CC3 IHC was shown for placebo and oxaliplatin groups. *Asterisk* indicated P < 0.05 for treatment group as compared to placebo, n = 5

The endpoint tumors were analyzed via IHC for proliferation (Ki-67), tumor vascularity (CD31) and programmed cell death (cleaved caspase 3, CC3). Ki-67 percent positive expression remained similar in all treatment groups, which suggested that proliferation was similar at the time of collection (Fig. 4a). CD31 percent positive expression was increased in oxaliplatin, erlotinib, and doxorubicin groups as compared to placebo control, but only erlotinib treatment was statistically significant (P = 0.044) (Fig. 4b). On the other hand, CC3 staining significantly increased only in the oxaliplatin treated tissue compared to placebo control (P = 0.019) (Fig. 4c, d).

### Oxaliplatin induces change in PACC morphology and re-expression of digestive enzymes

**Fig. 5:**
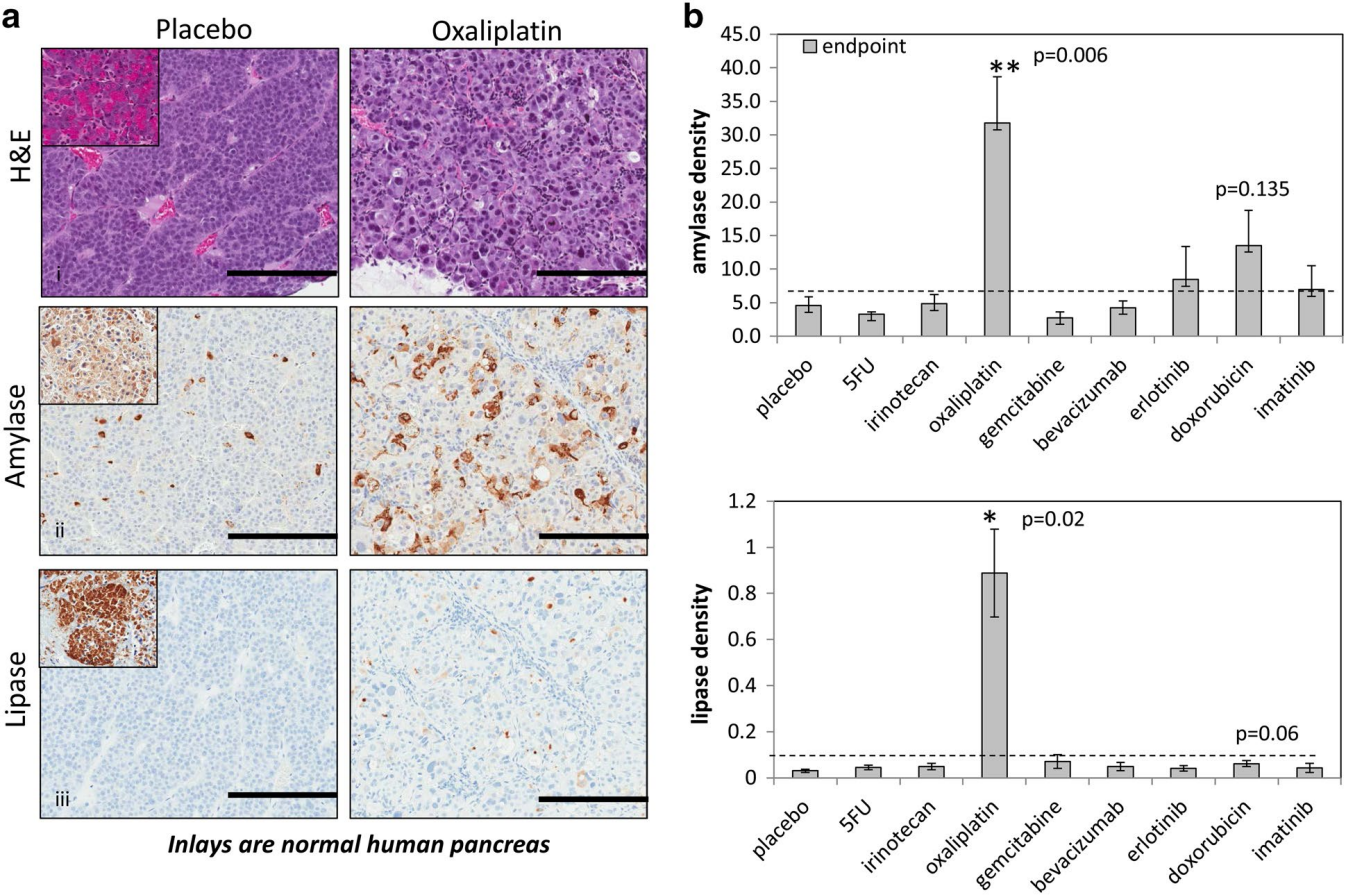
Oxaliplatin induces change in morphology and re-expression of digestive enzymes. **a** H&E depicted morphological changes and increased cell size in PA-018 after oxaliplatin treatment on endpoint tumor sections. There was also cytoplasmic retention of the digestive enzymes, amylase and lipase. Representative IHC was shown for placebo and oxaliplatin groups. Inlays are normal human pancreas. **b** Cytoplasmic amylase and lipase were quantitated by positive pixel count over area and plotted as mean ± standard deviation. **P < 0.01 for treatment group as compared to placebo, n = 5

In further examination of oxaliplatin’s effects, microscopic analysis showed that there was an increase in cytoplasmic content as well as cytoplasmic size as compared with placebo controls (Fig. 5a, panel i). Along with the histological changes, there was also a significant increase (P < 0.05) in cytoplasmic retention of amylase and lipase in the oxaliplatin treated tumors similar to normal pancreas (Fig. 5a panel ii, iii, b). We also noted that expression of collagen I was upregulated in certain portions of the oxaliplatin treated tissue when compared to placebo. Collagen I expression had been previously shown to be sparsely expressed around the borders of normal acinar cells and ducts [34, 35]. The Collagen I staining resembles the expression pattern of normal tissue in certain areas (Additional file 2: Figure S1).

### Gene analysis together with immunofluorescence (IF) and IHC indicates that the PACC patient and PA-018 PDTX model have a BRCA2 mutation

**Fig. 6:**
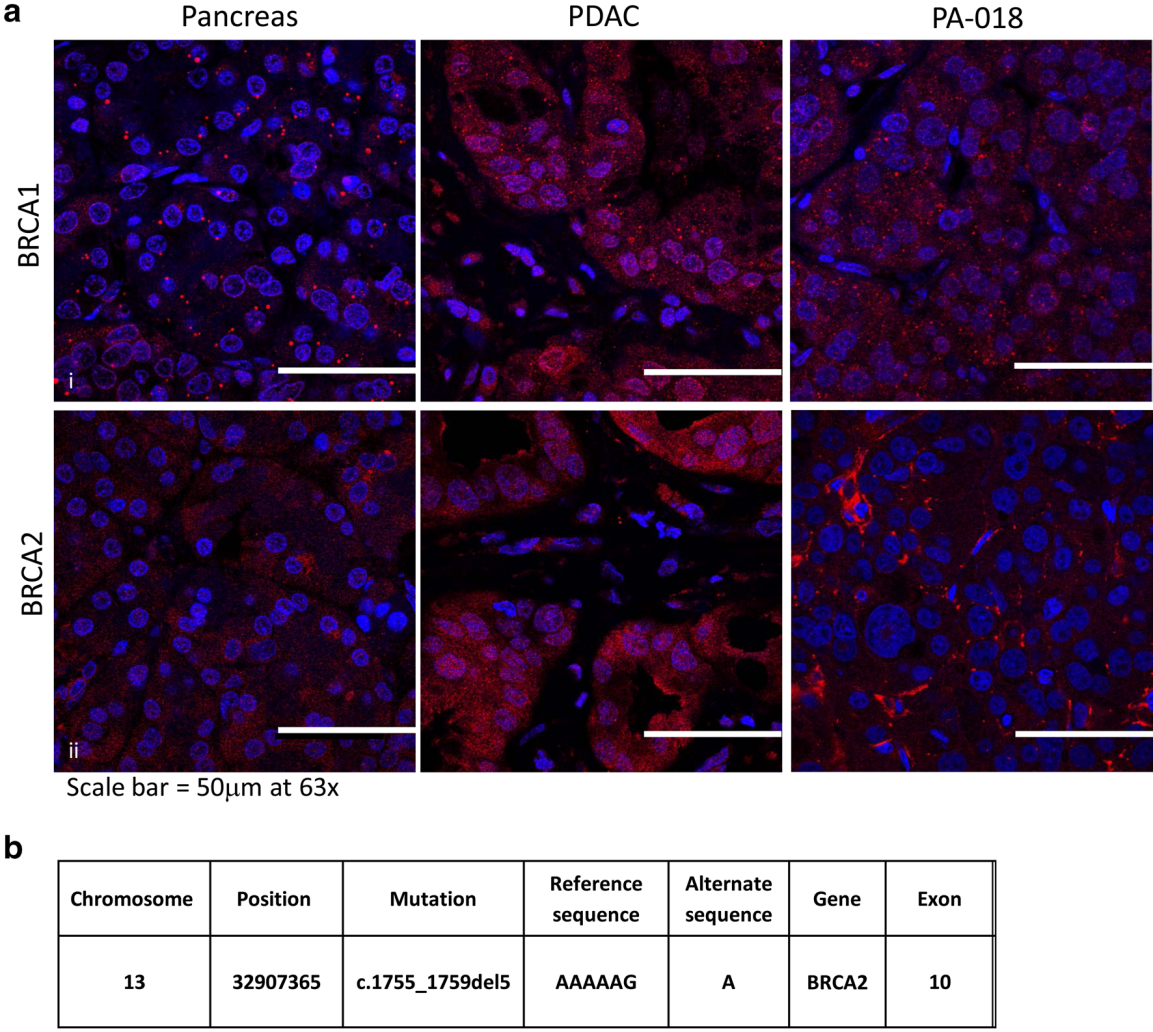
Immunofluorescence and genetic analysis indicates a BRCA2 mutation in the PA-018 PAAC model. **a** Immunofluorescence staining for BRCA1 and BRCA2. *Panel i* shows BRCA1 (*red*) expressed throughout the nucleus and cytoplasm in normal, PDAC, and the PAAC PDTX tissue. In *panel ii*, normal and PDAC tissue show co-localization of nuclear DAPI stain and BRCA2 but the PAAC PDTX tissue, does not have this co-localization, indicating that BRCA2 expression is confined to the cytoplasm. **b** Gene mutational analysis of BRCA2 confirms the presence a 5 base pair deletion on exon 10

An IF and IHC panel for BRCA1 and BRCA2 expression was performed on normal pancreas, pancreatic ductal adenocarcinoma (PDAC), and the PACC PDTX model. Both techniques demonstrate that nuclear BRCA1 is present in all tissue samples along with some cytoplasmic expression (Fig. 6a panel i; Additional file 3: Figure S2 panel i). Immunofluorescence shows that the PDTX model has no co-localization of BRCA2 and DAPI nuclear stain (Fig. 6a, panel ii). IHC on patient PACC tissue and its PDTX (PA-018) also show a lack of BRCA2 nuclear expression. Instead, only cytoplasmic expression in the islets and PACC tissue were observed (Additional file 3: Figure S2 panel ii). Mutational gene analysis of PA-018 PDX tissue was used to identify a 5 base pair deletion in BRCA2 (c.1755_1759del5) (Fig. 6b; Additional file 4: Figure S3). All sequence reads contained the mutated allele, indicating that there was no wild type BRCA2 allele present. This suggests a loss of heterozygosity (LOH) occurred in the tumor.

## Discussion

The rarity of pancreatic acinar cell carcinoma (PACC) has contributed to the lack of an effective standard treatment for this deadly disease. One way to understand a disease and test potential treatments is to have an in vivo laboratory model. To date, only a few recorded studies have been able to recapitulate PACC in mice. The oldest PACC model was formed by creating a transgenic mouse that expresses the transforming gene (T-antigen) of the SV40 virus under the control of elastase I, a pancreas specific promoter [36]. This model had been modified to express luciferase to record tumor burden and can be used to test chemotherapeutics [2]. Recently, another mouse model had been made by deleting a gene that blocked mTOR signaling [37]. The importance of mTOR was corroborated by the decreased tumor burden in both of these PACC mouse models when treated with rapamycin [2, 37]. While genetically engineered mouse models (GEMM)s was one approach to a successful model system, the generation of PDTXs hold unique benefits as well.

Within cancer, tumor heterogeneity exists and there may be multiple ways the same kind of cancer can arise [38, 39]. By creating PDTX models we can learn the similarities and differences between tumors that arose from different patients. We are also able to test tumors that have become chemoresistant to specific compounds and uncover possible alternatives [40, 41]. Thus, PDTXs provide representative signaling involved in disease progression and therapeutic responses that recapitulate those seen in patients [39, 42–47]. This is a report of the first fully characterized humanized model of PACC. Our in vivo model of human PACC can provide a novel means to further investigate the genetic and cellular mechanisms of this disease. Hence, we confirmed that the PDTX tumors were composed of human acinar cell carcinoma cells via STR profile, corroboration of biomarker expression seen in PACC patient tissues [15–18] and secretion of lipase into the bloodstream [14, 48] (Additional file 1: Table S1; Figs. 1, 3). With this data, we concluded that the PDTX tumor model (PA-018) was representative of our patient’s PACC, which provided the rationale for us to test multiple monotherapies based upon the patient’s past treatment history as well as gene array and protein expression data [20] (Fig. 2).

Imatinib showed little response in PA-018, this mimicked the mixed and only transient clinical response to imatinib in the patient [20]. Liposomal doxorubicin (Doxil) led to significant tumor shrinkage of multiple tumors in the liver of the patient, with a decrease in serum lipase and improvement in his quality of life [20]. Treatment was stopped due to known cardiac toxicity of doxorubicin and the patient’s cancer progressed. Similarly, the PDTX model demonstrated growth inhibition to liposomal doxorubicin but the overall outcome was not significant (P = 0.112) due to the tumor growing back after treatment ended (Fig. 2; Table 1). Bevacizumab, an angiogenesis inhibitor was briefly administered after the patient’s metastatic disease had progressed but due to symptoms of confusion, the therapy was discontinued before the efficacy of the drug could have been assessed. In this study, we saw a statistically significant time to end point tumor volume (TTE) of bevacizumab treated mice (P = 0.0413) (Fig. 2; Table 1). These data leaves open the possibility of bevacizumab as an effective chemotherapy.

We aimed to test standard treatments along with new rationally designed monotherapies. 5-FU and gemcitabine have both been clinically recommended chemotherapeutics for pancreatic cancer [49] and were added to this study to compare with drugs administered to the PA-018 patient. These two treatments were unable to maintain tumor reduction and were only better than imatinib (Fig. 2; Table 1). From our previous PACC study, we measured RNA levels of the PA-018 tumor biopsy and saw 2.5 fold elevated increase in TOPO1 which can be targeted by irinotecan [20]. As expected, irinotecan demonstrated a strong response while on therapy in PA-018, but off therapy the tumor continued to grow. EGFR expression was also elevated, so erlotinib was tested [20]. There were too many treatment related deaths to truly evaluate this data group. A lower dose of the drug may have to be considered in future exams. The most significant response in the PDTX model was observed following oxaliplatin treatment, which prevented tumor growth weeks after treatment cessation, leading to a durable response (Fig. 2; Table 1). Indeed, oxaliplatin was the only compound to significantly induce apoptosis in the PACC model (Fig. 4).

Like other platinum compounds, oxaliplatin inhibits DNA synthesis by forming both inter- and intra-strand cross links in DNA which prevent DNA replication and transcription, causing cell death [50]. Oxaliplatin is used for treatment of colorectal cancer when it is administered in combination with folinic acid and 5-fluorouracil, (FOLFOX) [51]. In addition, the standard of care for advanced pancreatic cancer is currently FOLFIRINOX (5-FU/folinic acid, irinotecan, and oxaliplatin) [52]. We pursued the beneficial effects of oxaliplatin treatment and observed that the PACC tissue retained amylase and lipase in the cytoplasm and stopped releasing lipase into the blood stream (Figs. 3b, 5). Serum lipase levels can be monitored in the blood as a measure of tumor burden with the expectation that the higher the value, the larger the tumor burden [20]. Serum samples from oxaliplatin treated mice were significantly decreased at day 15 of treatment as compared to the placebo controls (Fig. 3b). These data showed that it is not lipase production but lipase secretion that is indicative of disease, since normal pancreas accumulates lipase in the cytoplasm (Fig. 5a, panel iii) but barely secretes it [11]. By the end of the study, tissue morphology of oxaliplatin treated mice showed that the cancer cells became larger and resembled normal acinar cells more than PACC tissue (Fig. 5a panel i). Indeed, platin drugs have been reported to induce differentiation in cancer cells both in vitro and in vivo [53, 54]. Our data indicates that oxaliplatin inhibits PACC tumor growth by inducing re-differentiation and promoting apoptosis (Figs. 4c, d, 5). Next we assessed why our PACC PDTX model responded well to oxaliplatin.

Previous literature has shown that the presence of BRCA mutations (mutations in a gene involved in DNA repair) in both breast and ovarian cancers are associated with increased sensitivity to platinum compounds [55–57]. This sensitivity has also been shown in ovarian tumors with either germline or sporadic somatic mutations [57]. Furthermore, within the small subset of PDAC patients carrying germline BRCA gene mutations, those treated with platinum compounds had an increase in overall survival [58]. These findings are of particular interest because genomic profiling on 44 PACC patient tumors uncovered DNA repair mutations in 45 % of the samples [19]. In our PDTX model, BRCA1 was mostly localized in the nucleus, while BRCA2 was mostly localized in the cytoplasm and absent from the nucleus, which suggests that the protein is truncated and lost its nuclear localization sequence [59, 60] (Fig. 6a; Additional file 3: Figure S2). The BRCA2 truncation prevents the cell from entering the nucleus and taking part in the DNA homologous recombination repair pathway [57]. Mutational gene analysis of BRCA2 confirms that a 5 base pair deletion on exon 10 (c.1755_1759del5) was present in our PDX model (Fig. 6b). BRCA2 deletions have frequently been associated with deleterious protein truncations [61, 62]. The same gene deletion (referred by the Breast Cancer Information Core as 1983del5) has even been reported as a familial BRCA2 mutation [63]. Using this evidence, we concluded that a BRCA2 truncation mutation may be one potential reason our PACC model was sensitive to oxaliplatin induced cell death. Indeed, previous literature has reported case of a PACC patient with a hereditary BRCA2 mutation. The individual was on a combination of gemcitabine and oxaliplatin, which prolonged their survival, several years longer than the average life expectancy of PACC patients [64]. Overall, previous findings together with our data suggest that oxaliplatin may be beneficial to PACC patients whose tumors carry DNA repair mutations, such as BRCA2.

## Conclusions

In summary, it was supported that this newly characterized PACC PDTX model first recapitulated the monotherapy response seen in its matching patient tissue and in the process secondly discovered that oxaliplatin was very effective as a monotherapy in this PACC model. Because of the rarity of PACC, only a handful of retrospective articles and case reports describing experimental therapy have been published. Our findings involving oxaliplatin in this PDTX model warrant further evaluation for the management of pancreatic acinar cell carcinoma. As the first and only preclinical human PACC derived model, the scientific community can use it to better understand the pathobiology of this disease.

## Additional files

10.1186/s12967-016-0875-z STR profile comparison of patient tumor biopsy and PA-018 PDTX. Short tandem repeat (STR) analysis showed that the genetic signature of our PDTX tumor (passage 5) closely matched the signature of the patient. The DNA that was used for comparison was from a sample taken 3 years prior to the PA-018 biopsy due to unavailable tissue. Five allele drop out events were noted to have occurred.
10.1186/s12967-016-0875-z Expression Collagen I on Normal Pancreas and PACC. Collagen I borders acinar cell clusters and normal ductal structures. These borders were usually minimal in PA-018 unless treated with oxaliplatin, which restored expression of collagen I in certain regions of the PDTX tissue.
10.1186/s12967-016-0875-z IHC suggests that patient and PA-018 PDTX model have a truncating BRCA2 mutation. IHC panel for BRCA1 and BRCA2 expression was performed on normal pancreas, pancreatic ductal adenocarcinoma (PDAC), patient PACC and its PDTX (PA-018). Panel i. BRCA1 was both nuclear and cytoplasmic in all samples (panel i). Panel ii. Nuclear expression of BRCA2 was absent in the PACC tissue of both the patient’s original tumor and PA-018 as compared to PDAC. Only cytoplasmic expression in the islets of normal tissue and PACC tissue was observed.
10.1186/s12967-016-0875-z Integrated genomic viewer (IGV) of BRCA2 gene. IGV displays genomic data of the PA-018 PAAC PDTX model. Chromosome 13 (Chr 13) is shown and 5bp deletions are found after position 32907365 (c.1755_1759del5), this region resides on exon 10 of BRCA2. The bottom of the image shows the nucleotides and amino acids that correspond to the reference sequence of the BRCA2 gene and protein.

## Abbreviations

PACC: pancreatic acinar cell carcinoma
PDTX: patient derived tumor xenograft
CEA: carcinoembryonic antigen
CK18: cytokeratin 18
BCL10: B cell lymphoma/leukemia 10
CR: Charles Rivers
5-FU: 5-Fluorouracil
D5W: 5 % dextrose in water
TTE: time-to-endpoint
TR: treatment-related
NTR: non-treatment-related
TGD: tumor growth delay
BW: body weight
MTD: maximum tolerated dose
IHC: immunohistochemistry
TMA: tissue microarray
NSE: neuron specific enolase
CgA: chromogranin A
CK19: cytokeratin 19
CC3: cleaved caspase-3
STR: short tandem repeat
CK19: cytokeratin 19
FOLFOX: folinic acid and 5-fluorouracil
FOLFIRINOX: 5-FU/folinic acid, irinotecan, and oxaliplatin
