# Supplementary material for: Novel patient-derived xenograft mouse model for pancreatic acinar cell carcinoma demonstrates single agent activity of oxaliplatin
Source: J Transl Med. 2016 May 10;14:129. doi: 10.1186/s12967-016-0875-z (PMC4862141; doi:10.1186/s12967-016-0875-z)
Supplement: Supplementary file 1 — 10.1186/s12967-016-0875-z STR profile comparison of patient tumor biopsy and PA-018 PDTX. Short tandem repeat (STR) analysis showed that the genetic signature of our PDTX tumor (passage 5) closely matched the signature of the patient. The DNA that was used for comparison was from a sample taken 3 years prior to the PA-018 biopsy due to unavailable tissue. Five allele drop out events were noted to have occurred. [file 12967_2016_875_MOESM1_ESM.docx]

Table S1

|  | **AMEL** | **D5S818** | **D13S317** | **D7S820** | **D16S539** | **vWA** | **TH01** | **TPOX** | **CSF1PO** |
| --- | --- | --- | --- | --- | --- | --- | --- | --- | --- |
| **patient** | XY | 12 | 8,9 | 9,10 | 8,10 | 16,17 | 6,9.3 | 8,11 | 11,12 |
| **PA-018** | XY | 12 | 9 | 9,10 | 10 | 16,17 | 9.3 | 8 | 12 |
