# Supplementary figures and images for: Novel patient-derived xenograft mouse model for pancreatic acinar cell carcinoma demonstrates single agent activity of oxaliplatin
Source: J Transl Med. 2016 May 10;14:129. doi: 10.1186/s12967-016-0875-z (PMC4862141; doi:10.1186/s12967-016-0875-z)

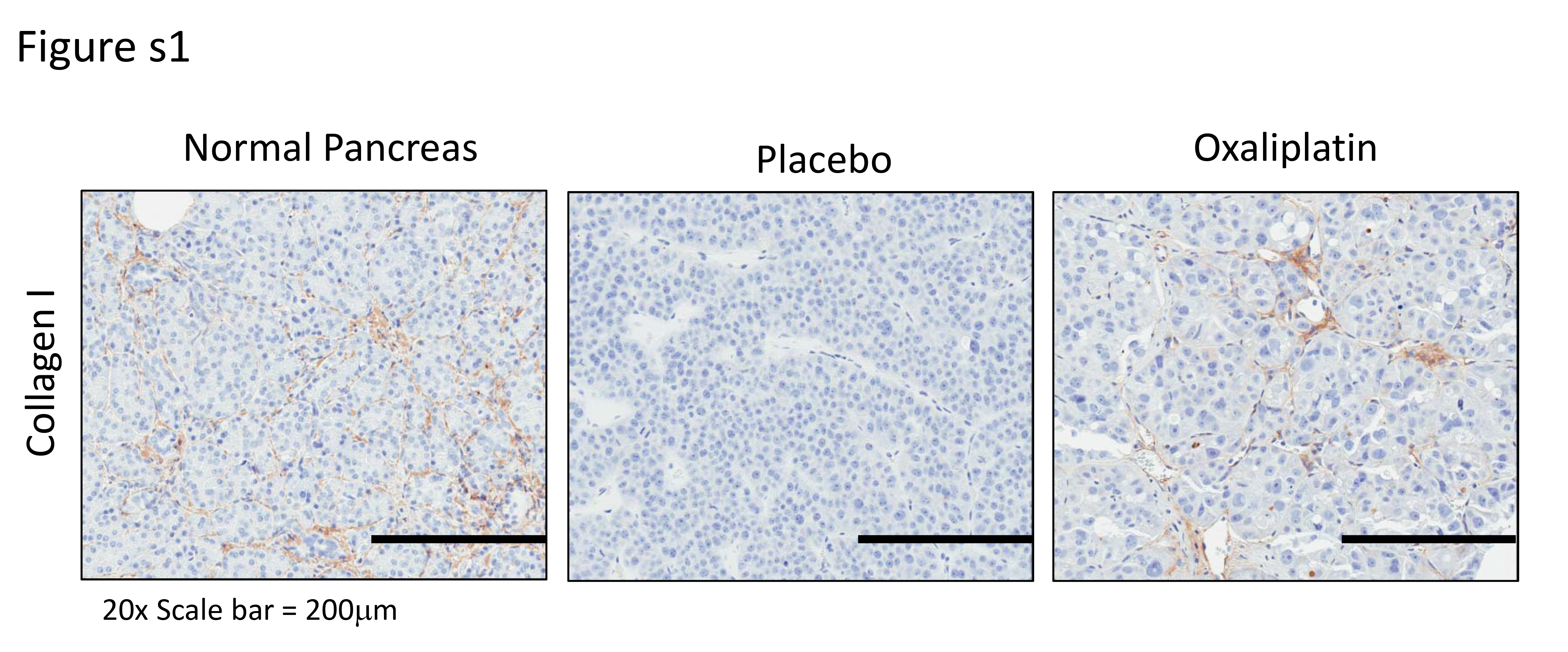

Supplement: Supplementary file 2 — 10.1186/s12967-016-0875-z Expression Collagen I on Normal Pancreas and PACC. Collagen I borders acinar cell clusters and normal ductal structures. These borders were usually minimal in PA-018 unless treated with oxaliplatin, which restored expression of collagen I in certain regions of the PDTX tissue. [file 12967_2016_875_MOESM2_ESM.tif]

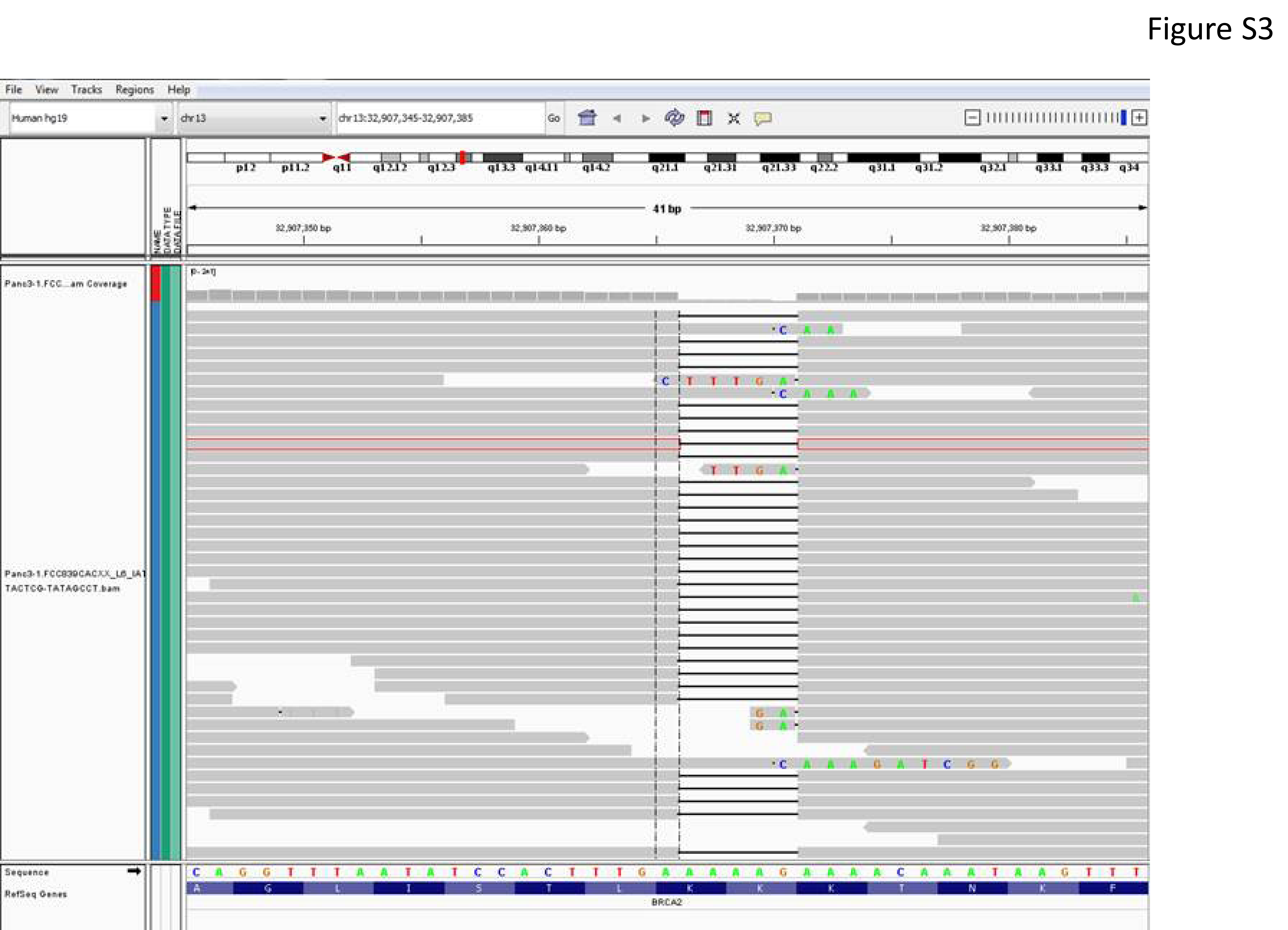

Supplement: Supplementary file 4 — 10.1186/s12967-016-0875-z Integrated genomic viewer (IGV) of BRCA2 gene. IGV displays genomic data of the PA-018 PAAC PDTX model. Chromosome 13 (Chr 13) is shown and 5bp deletions are found after position 32907365 (c.1755_1759del5), this region resides on exon 10 of BRCA2. The bottom of the image shows the nucleotides and amino acids that correspond to the reference sequence of the BRCA2 gene and protein. [file 12967_2016_875_MOESM4_ESM.tif]
